# The role of SUMOylation in the neurovascular dysfunction after acquired brain injury

**DOI:** 10.3389/fphar.2023.1125662

**Published:** 2023-03-22

**Authors:** Pengren Luo, Lin Li, Jiashang Huang, Deqiang Mao, Silong Lou, Jian Ruan, Jie Chen, Ronghua Tang, You Shi, Shuai Zhou, Haifeng Yang

**Affiliations:** ^1^ Department of Neuro-Oncology, Chongqing University Cancer Hospital, Chongqing, China; ^2^ Department of Neurosurgery, The Affiliated Hospital of Kunming University of Science and Technology, Kunming, China

**Keywords:** acquired brain injury, neurovascular dysfunction, small ubiquitin-like modifier, SUMO, post-translational modifications

## Abstract

Acquired brain injury (ABI) is the most common disease of the nervous system, involving complex pathological processes, which often leads to a series of nervous system disorders. The structural destruction and dysfunction of the Neurovascular Unit (NVU) are prominent features of ABI. Therefore, understanding the molecular mechanism underlying NVU destruction and its reconstruction is the key to the treatment of ABI. SUMOylation is a protein post-translational modification (PTM), which can degrade and stabilize the substrate dynamically, thus playing an important role in regulating protein expression and biological signal transduction. Understanding the regulatory mechanism of SUMOylation can clarify the molecular mechanism of the occurrence and development of neurovascular dysfunction after ABI and is expected to provide a theoretical basis for the development of potential treatment strategies. This article reviews the role of SUMOylation in vascular events related to ABI, including NVU dysfunction and vascular remodeling, and puts forward therapeutic prospects.

## 1 Introduction

### 1.1 Neurovascular dysfunction after acquired brain injury

#### 1.1.1 Pathology of ABI

Acquired Brain Injury (ABI) typically is classified into the two following types: traumatic brain injury (TBI) and non-TBI (non-TBI) (Bruns and Hauser, 2003), with the former having the highest incidence among neurological diseases (Maas et al., 2022). The causes of TBI include traffic accidents, violence, and other accidents^1^. The occurrence of non-TBI is related to stroke, neoplasm, infection, inflammation, anoxia, alcohol consumption, and drug use (Goldman et al., 2022). ABI leaves patients with physical and psychological sequelae, making it difficult for them to reintegrate into society, resulting in a great social burden (Menon and Bryant, 2019). ABI can cause vascular structure damage, dysfunction of the neurovascular unit (NVU), destruction of the blood-brain barrier (BBB), brain edema, activation of immunoreactive cells, the release of immune mediators, oxidative stress, mitochondrial and metabolic dysfunctions, and changes in cerebral blood flow (CBF) (Greve and Zink, 2009; Zibara et al., 2019). These eventually lead to headaches, cognitive changes, emotional anxiety, and a series of neurological disorders (Bedaso et al., 2018; Larsen et al., 2019; Chan and Woldeamanuel, 2020).

The primary injury of ABI is of the mechanical kind and includes the destruction of neurons, glia, and vascular structures. It is irreversible. However, secondary injury occurs over time and initiates multiple signaling cascades, which can change cell function and cause cell death, including oxidative stress, excitotoxicity, mitochondrial dysfunction, and inflammation (Lozano et al., 2015). Within minutes of ABI, astrocytes, and microglia begin to secrete pro-inflammatory factors (including interleukin-6, interleukin-1, tumor necrosis factor, *etc.*), causing local inflammation (Ghirnikar et al., 1998; Ekmark-Lewen et al., 2010; Homsi et al., 2010). These inflammatory factors actively recruit immune cells and glial cells, thus aggravating the occurrence of inflammation (Lossinsky and Shivers, 2004). Some studies have shown that microglia can maintain a state of enhanced inflammation for several months after ABI (Witcher et al., 2015). The inflammatory responses after ABI include the activation of immune cells in the central nervous system (CNS) and infiltration of peripheral immune cells through the BBB, which is mediated by a variety of inflammatory cytokines. The dysfunction of NVU and BBB caused by ABI allows activated white blood cells to migrate to the damaged brain parenchyma, which is also promoted by the upregulation of cell adhesion molecules. Activated white blood cells, microglia, and astrocytes produce ROS and inflammatory molecules, like cytokines and chemokines, leading to demyelination and destruction of the axonal cytoskeleton (Goldman et al., 2022). Over time, inflammatory events lead to brain edema and increased intracranial pressure (McGinn and Povlishock, 2016). ABI is characterized by the crossover and fusion of highly related neuropathological reactions, wherein severe and persistent inflammatory reactions may cause further deterioration. However, although neuroinflammation aggravates brain damage in the early stages of ABI, inflammation may also promote angiogenesis and neurogenesis at a later stage (Candelario-Jalil et al., 2022).

**FIGURE 1:**
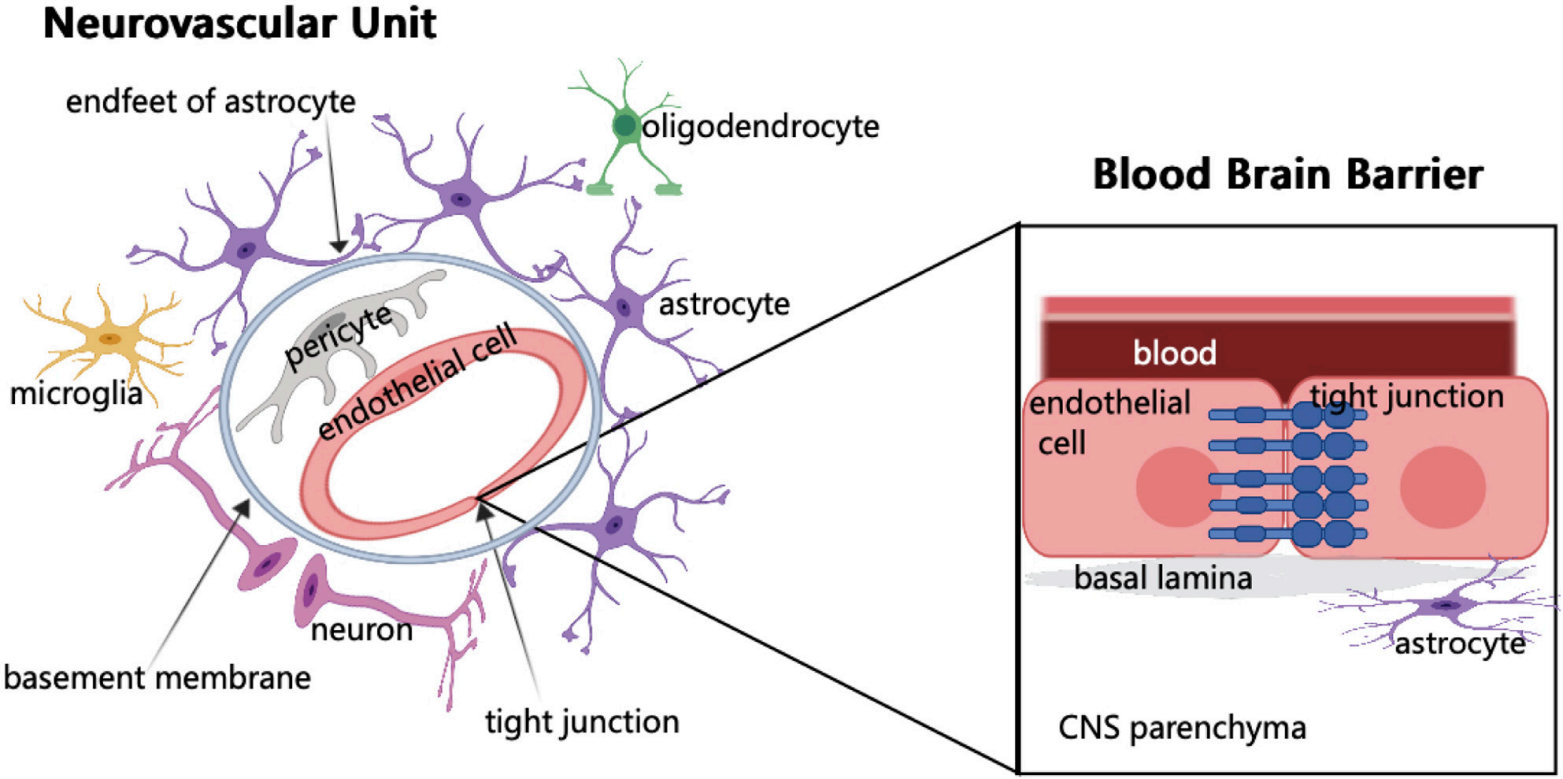
Schematic of neurovascular unit and blood brain barrier components. In NVU, endothelial cells are surrounded by pericytes, which are embedded in the basement membrane and surrounded by the endfeet of astrocytes. The synaptic connection between astrocytes and neurons regulates neurons. When brain tissue is injured, microglia respond first. In addition, NVU consists of few oligodendrocytes. The main component of BBB is endothelial cells. The junctions between endothelial cells are involved in regulating cell bypass permeability.

The vascular network of the brain originates from the internal carotid artery and goes deep into the brain. The maintenance of normal brain function requires the integrity of brain structure, normal synaptic activity, and smooth transmission of information. However, these require the coordination of different cells in the NVU and the structural integrity of BBB (Zhao et al., 2015) (Figure 1). In NVU, endothelial cells (ECs) are part of BBB. These ECs are related to several substrate-specific transport systems and can control the transport of nutrients, metabolites, and some essential molecules (Zhao et al., 2015). The concept of NVU regards the interaction among neurons, glial cells, and cerebral vessels as a whole, and promotes the previous treatment singly, such as neuron protection and vascular remodeling to the level of protection and repair of NVU, bringing new opportunities for research on the treatment of ABI.

#### 1.1.2 BBB damage and NVU dysfunction

BBB is formed by ECs of the capillary wall, astrocyte end-feet ensheathing the capillary, and pericytes embedded in the capillary basement membrane (Ballabh et al., 2004). The characteristics of BBB are mainly determined by the connection complex between brain ECs, including tight, adherens, and gap junctions. Although it has a strong similarity with the epithelium, the brain endothelial junction complex has a special structure and unique pattern of protein expression (Stamatovic et al., 2016). BBB is a highly selective physical barrier that strictly controls the microenvironment of brain cells. As the connection between ECs effectively limits the permeability of the adjacent cells, and these cells lack phagocytic vesicles, thus limiting the transport across the fine cytoplasm, the molecules are transported to CNS by active transport or passive diffusion (Daneman and Prat, 2015). Vascular ECs not only regulate the selectivity and permeability of BBB but also CBF, angiogenesis, and neuronal development (Iadecola, 2017). Although the barrier of the cerebrovascular system mainly comprises ECs, these are not functioning alone but as a part of the NVU, resulting in a complex molecular crosstalk network with different cellular elements in the NVU (Neuwelt et al., 2011). The formation and stability of cerebral vessels and BBB depend on the expression of growth factors, guiding molecules, and signal pathways (Andreone et al., 2015; Walchli et al., 2015). The function of BBB is to limit the circulation of harmful substances and blood cells in the blood in cerebral vessels which cannot enter the brain parenchyma (Winkler et al., 2011). Another important role of BBB is to reduce cross-cell permeability (Zhao et al., 2015). ABI can cause BBB damage, and various components in the blood will invade the brain tissue, resulting in cell infiltration and damage (Zhao et al., 2015; Montagne et al., 2017). BBB can cause an imbalance in CBF (Iadecola, 2017; Kisler et al., 2017), initially resulting in the disorder of the normal physiological function of the nervous system. Therefore, as a structure connecting the central nervous system and systemic circulation, BBB is the key to maintaining the homeostasis of the nervous system.

The concept of NVU emerged in 2001 to emphasize the relationship between the microvascular system and structural integrity and functional maintenance of brain cells, along with the coordinated response between the two in the event of a brain injury (Iadecola, 2017). NVU includes ECs, basement membranes, pericytes, astrocytes, microglia, and neurons, as well as oligodendrocytes (Sweeney et al., 2019). Among the many components, microglia can promote endothelial repair, thus maintaining endothelial integrity (Thurgur and Pinteaux, 2019). The function of neurons is to regulate the activity of enzymes to meet the metabolic needs of the brain (McConnell et al., 2017). In the vascular system of the brain, there is a three-dimensional vascular network comprising pial arteries, capillaries, ascending venules, and leptomeningeal veins. NVU exists in this vascular network (Blinder et al., 2013). This three-dimensional vascular network can enter the deep part of the brain, nourish the neurons, and transport the metabolites away (Kisler et al., 2017). The maintenance of normal brain function lies not only in the connection between neurons but also in the coordination between different components of the NVU (Lo and Rosenberg, 2009). NVU is indispensable for the normal maintenance of the function of the CNS. If NVU is destroyed, various cerebrovascular diseases can occur, eventually leading to nervous system dysfunction. However, NVU disorders are common in ABI, and the causal relationship between NVU dysfunction and diseases is not completely clear (McConnell et al., 2019).

#### 1.1.3 New vessel formation after ABI

The main damages of ABI to cerebral vessels include hemorrhage, edema, abnormal CBF and destruction of BBB (Salehi et al., 2017). Several studies have shown that after cerebral vascular injury, it will try to repair it (Morgan et al., 2007; Park et al., 2009). Related studies have shown that the cerebrovascular system begins to repair within two to 3 weeks of damage, including not only larger blood vessels, but also smaller blood vessels, such as capillaries (Park et al., 2009; Siddiq et al., 2012). After ABI, the abnormal structure of vascular wall, swelling and apoptosis of endothelial cells and degradation of extracellular matrix could be seen under electron microscope (Danaila et al., 2013; Jullienne et al., 2014). The formation of new blood vessels after ABI is crucial to the recovery of nerve function. Existing literature shows that angiogenesis and vasculogenesis are two main mechanisms occurring after brain injury (Salehi et al., 2017). These complex but different processes play an important role in repairing the damaged vascular system after brain injury (Salehi et al., 2017). Angiogenesis refers to the formation of new blood vessels from the existing vascular system, while vasculogenesis is the occurrence of neovascularization.

Hypoxia caused by interruption of blood flow after ABI is the main cause of triggering angiogenesis (Gajavelli et al., 2015). This may be related to the expression regulation of hypoxia-related molecules, such as HIF-1 α (Anderson et al., 2009). Vascular endothelial cells are activated after ABI (Balabanov et al., 2001). Endothelial cells form vascular sprouts and migrate to hypoxia (Van Hove and Benoit, 2015). With the accumulation of pericytes, the damaged blood vessels were repaired (Hirota and Semenza, 2006). The occurrence of vasculogenesis mainly comes from endothelial progenitor cells, so this process is actually initiated in the bone marrow (Asahara et al., 1999). After ABI, endothelial progenitor cells peak within a week after a brief decrease, and play a role after maturation in the blood (Gong et al., 2011; Liu et al., 2011). In addition, endothelial progenitor cells also release some factors, which can promote the proliferation and migration of ECs and promote vasculogenesis (Ziegelhoeffer et al., 2004; Urbich et al., 2005).

After the occurrence of ABI, timely and effective intervention can restore the function of NVU to varying degrees. Angiogenesis and vascular remodeling can rebuild the BBB, protect brain tissue from further damage, provide necessary nutrition, and play a key role in nerve repair (Esquiva et al., 2018).

### 1.2 Protein post-translational modifications

Proteins play an important role in most biological processes. Their functions are regulated by several protein PTMs. PTMs refer to amino acid chain or terminal covalent enzyme modification, a reversible process that can affect the activity, localization, interaction, and function of target proteins (Wang et al., 2014). PTMs are the core of many cellular signaling events. Usually, PTMs can work alone or cooperatively. The crosstalk between PTMs often affects the physiological and pathological processes of cells through fine-tuning (Vu et al., 2018). PTMs usually occur in the C- or N-terminal of amino acid side chains of proteins, including the addition of chemical or functional groups, polypeptide chains, complex molecules, and modification of amino acids, depending on the type of PTMs (Xu et al., 2018). More than 450 kinds of PTMs have been found (Venne et al., 2014), including acetylation, ubiquitination, methylation, glycosylation, phosphorylation, and SUMOylation (Zheng and Shabek, 2017). Ubiquitin has lysine residues that act on the target protein and complete mono-ubiquitination or poly-ubiquitination under the action of ligase, binding enzyme, and activating enzyme. This process can be reversed by deubiquitinase (Deng et al., 2020). Phosphorylation mainly acts on serine, threonine, and tyrosine residues of target proteins (Olsen et al., 2006). Methylation mainly occurs on lysine and arginine residues. Lysine can be monomethylated, demethylated, or trimethylated by lysine methyltransferases; arginine can be monomethylated or dimethylated by arginine methyltransferases (Zhang et al., 2012; Yang and Bedford, 2013). Glycosylation can be linked to the side chain by oligosaccharide transferase by an amide bond (Moremen et al., 2012). As a PTM, SUMOylation has received more attention in recent years, because it is necessary to maintain genome integrity, molecular signal transduction, transcriptional regulation, and gene expression (Han et al., 2018). When PTMs in the cellular environment are dysfunctional, conformational changes of related proteins, imbalance of enzyme activity, abnormal protein folding, and production of toxic metabolites occur, all eventually leading to the disease state (Stram and Payne, 2016).

### 1.3 SUMOylation

SUMOylation is a common PTM of proteins. Small ubiquitin-like modifier (SUMO), similar to ubiquitin in structure, is an important protein discovered after ubiquitin (Yeh et al., 2000; Garcia-Rodriguez et al., 2016). Although the structure of SUMO is similar to that of ubiquitin, its cellular functions differ. The main function of ubiquitination is to degrade substrate proteins, while SUMOylation chiefly regulates cell localization, protein transcription, protein interaction, and DNA repair processes (Garcia-Rodriguez et al., 2016; Han et al., 2018). When SUMO binds to a protein, it can change the location, conformation, activity, and gene expression of the target protein, thus affecting many physiological and pathological processes (Chymkowitch et al., 2015).

Five SUMO homologous genes, named SUMO 1–5 are expressed in humans (Huang et al., 2004; Liang et al., 2016). SUMO 1–3 is widely expressed and participates in the modifications of thousands of proteins (Hendriks et al., 2017; Hendriks et al., 2018). The homology between SUMO2 and SUMO3 is very high, up to 97%. Therefore, the two cannot be completely distinguished and are usually termed SUMO2/3. They exist in various locations in the cell in a non-conjugated form and can be attached to the target protein after stimulating correspondingly (Enserink, 2015; Niskanen and Palvimo, 2017; Bernstock et al., 2018a). The expression of SUMO4–5 is limited, and it is not clear whether these can bind to the target protein (Celen and Sahin, 2020). In the SUMO family, SUMO2 has the highest abundance compared to the other members, so the lack of SUMO2 cannot be compensated by other analogs. In contrast, the functions of SUMO1 and SUMO3 can be compensated by the other analogs (Wang et al., 2014).

**FIGURE 2:**
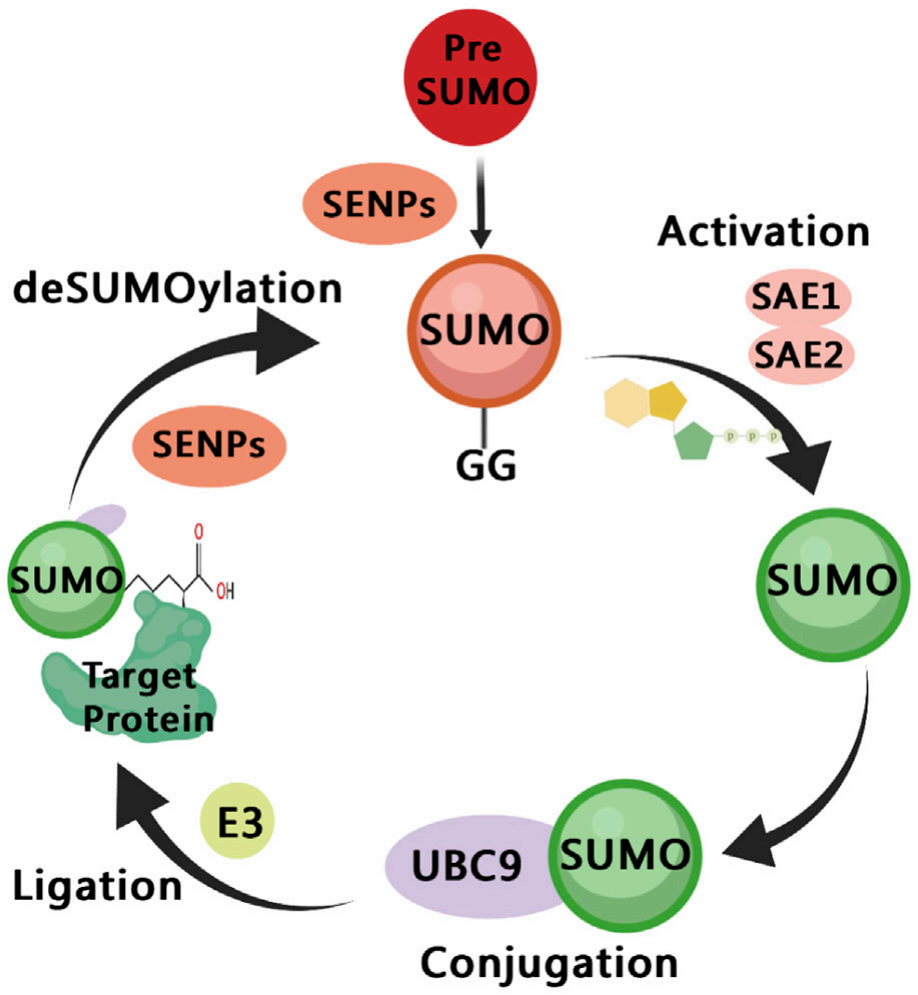
A diagram of the SUMOylation pathway. The precursor of small ubiquitin-like modifier (SUMO) are cleaved by SENPs, reveal the C-terminal diglycine motif (Maturation); It is then activated by the E1 enzyme SAE1/SAE2, which depends on ATP (Activation); The activated SUMO is transferred to UBC9 (Conjugation); SUMO is connected to the side chain of the specific lysine residue of the target protein, and this process involves E3 ligase (Ligation); SUMO is dissociated from the target protein, and the free SUMO can participate in the next catalytic cycle, which is regulated by SENPs (deSUMOylation).

SUMOylation is a dynamic and reversible process (Figure 2), similar to the cascade reaction of ubiquitin enzymes (Gill, 2004). The SUMO subtype binds to the target protein through the three following steps: activation, heterodimer E1 enzyme (SAE1, SAE2), binding, E2 enzyme (UBE2I/UBC9), substrate modification, and E2 and E3 protein ligase interaction (Lv et al., 2018). The SUMOylation pathway is regulated by several enzymes, among which proteases and ligases are the most important regulatory modes. The most studied proteases and ligases are of the SENP and PIAS families, respectively (Lara-Urena et al., 2022). The proteases involved in SUMOylation modification are specific, and the most studied proteases are SENP1-3 and SENP5-7 of the SENP family (Kunz et al., 2018). SUMO-specific peptidases (SENP1, 2, 3, 5, 6, and 7) catalyze the deSUMOylation reaction, thus dissociating lysine residues of target proteins from SUMO (Rawlings et al., 2019). PIAS family is mainly involved in DNA binding and transcriptional regulation (Rabellino et al., 2017).

Many studies have shown that abnormal regulation of SUMOylation is associated with a variety of diseases, including heart and neurodegenerative disorders (Da Silva-Ferrada et al., 2016; Mun et al., 2016). The balance of SUMOylation plays an important role in cardiac development, metabolism, and stress (Gupta et al., 2016; Gupta and Robbins, 2016; Liu et al., 2017). Ubc9-mediated SUMOylation can effectively reduce the incidence of proteotoxic heart diseases (Gupta et al., 2016). Moreover, therapeutic targets for cardiac fibrosis and heart failure are also closely related to Ubc9 (Liu et al., 2017). In neurodegenerative diseases, SUMOylation causes abnormal accumulation of HTT protein, leading to Huntington’s disease, which is related to abnormal degradation of the ubiquitin-proteasome pathway (Steffan et al., 2004). SUMOylation is also closely related to Alzheimer’s disease, which is related to the abnormal expression of amyloid- β and tau protein (Yang et al., 2017). Dysfunctional SUMOylation also plays an important role in cancer. The upregulation of SUMO binding enzyme increases SUMOylated proteins, leading to poor prognosis (Moschos et al., 2007; Chen et al., 2011; Zhang et al., 2013; Seeler and Dejean, 2017). In several cancers, the abnormal expression of SENPs may promote their progression (Ding et al., 2008; Bawa-Khalfe and Yeh, 2010; Sun et al., 2013). SUMOylation plays an important role in the study of drug resistance in hepatocellular carcinoma (Qin et al., 2014). Several therapeutic targets against cancer-related to SUMOylation are being studied. In short, the imbalance between SUMOylation and deSUMOylation is related to the occurrence and development of many diseases. Therefore, it is meaningful to study the relationship between SUMOylation and various diseases.

This review focuses on neurovascular dysfunction after ABI, with particular emphasis on the correlation between SUMOylation and neurovascular dysfunction. Actively examining potential molecular targets is needed and this review discusses the possibilities for the development of a new treatment for ABI.

## 2 Regulatory effects of SUMOylation on the components of NVU

### 2.1 The regulatory effects of SUMOylation on ECs

ECs in cerebral vessels are the core components of BBB, and their morphology and function are different from those of peripheral ECs (Daneman and Prat, 2015). The ECs in the cerebral vessels have more mitochondria than the peripheral ECs, consuming more energy to drive the ion gradient, which is important for enzyme activity (Daneman and Prat, 2015). ECs do not have small transcellular pores that allow free diffusion, so these can prevent rapid molecular exchange between blood and brain tissues (Pandit et al., 2020). The tight connection between ECs can completely close the gap between them to form continuous blood vessels (Tietz and Engelhardt, 2015). It also promotes the transmission of information between neurons and glial cells (Wang et al., 2015). Normal ECs also play an important role in regulating thrombosis and vasodilation (Findlay et al., 1989). ECs can secrete vasoactive factors to control vasodilation and contraction and vasoconstrictors, thus maintaining the balance in CBF (Sandoo et al., 2010). Endothelial cell dysfunction (ECD) destroys the integrity of BBB, leading to changes in CBF, eventually causing functional damage and degradation of the NVU (Iadecola, 2017), and mediating the occurrence and progression of ABI. The essence of ECD is a change in their functional phenotype, such as a deficiency in nitric oxide (NO), senescence of ECs, and increased expression of endothelial-leukocyte adhesion molecules, which are the basis for thrombosis and inflammation (Gimbrone and Garcia-Cardena, 2016; Anand et al., 2018; Harding et al., 2019). Given the importance of ECD in ABI, it remains the focus of future research.

#### 2.1.1 EC senescence and apoptosis

Vascular aging can lead to many cardiovascular and cerebrovascular diseases, including stroke, hypertension, and coronary heart disease, and EC aging is an important characteristic of vascular aging (Ungvari et al., 2018). Reactive oxygen species (ROS) produced by mitochondrial dysfunction can activate microglia continuously (Faas and de Vos, 2020; Riley and Tait, 2020). The increase in ROS in microglia leads to cell death and neuroinflammation (Chiurazzi et al., 2020). Mature mitochondrial thioredoxin 2 (Trx2) is an important redox protein, which can eliminate ROS in time and slow down the senescence of VECs (Papaconstantinou, 2019). However, the precursor of Trx2 (PreTrx2) cannot perform the function of scavenging ROS. SUMOylation of Trx2 can reduce the accumulation of PreTrx2, promote the formation of Trx2, and effectively inhibit the senescence of ECs induced by ROS (Chen et al., 2019). Trx family of proteins reportedly helps reduce ischemic ABI (Otero-Losada et al., 2019). This may be related to the fact that cytoplasmic Trx1 and mitochondrial Trx2 can inhibit the production of free radicals, reduce oxidative stress, and mitochondria-dependent apoptosis (Chiueh et al., 2005). A previous study suggests that increased expression of Trx2 exerts a neuroprotective effect (Lee et al., 2017). A series of studies have obtained useful results, and the effect of Trx2’s SUMOylation on ABI is worthy of further study.

An animal study on pigs showed that increased expression of SUMO1 in the porcine arterial ECs could protect ECs, reduce EC apoptosis, and increase EC formation. Mechanistic studies have shown that SUMO1 promotes angiogenesis by regulating MMP13 expression and the JAK2/STAT5 signaling pathway, and similar results were obtained using mouse models (Yang et al., 2013). The effect of SUMOylation on endothelial cell apoptosis and senescence is not only that, we need to invest more research to prove it.

#### 2.1.2 EC inflammation

GATA2, a transcription factor that regulates the expression of EC adhesion molecules, can be modified by SUMOylation, During inflammation, SENP1 promotes the deSUMOylation of GATA2 in ECs, increasing the stability of GATA2, thus finally aggravating endothelial inflammation (Qiu et al., 2017). NLRP3 inflammatory bodies can activate IL-1 β and increase the intensity of inflammation (Wang et al., 2017). Several studies have shown that the initiation and activation of NLRP3 are significantly upregulated after ABI (O'Brien et al., 2020). SUMOylation deletion of NLRP3 can increase the expression of IL-1 β, while the deletion of SENP7 downregulates the expression of IL-1 β, indicating that NLRP3 can inhibit the progression of inflammation through SUMOylation (Barry et al., 2018). Here, SUMOylation seems to show anti-inflammatory effects.

Angiotensin II (Ang II) is a key molecule in the renin-angiotensin system that regulates blood pressure, which can promote the production of ROS, apoptosis, and inflammation in ECs (Benigni et al., 2010). The activation of transcription factor 3 (ATF3), an adaptive reactive protein, and its abnormal expression lead to atherosclerosis (Forrester et al., 2018). Silencing ATF3 can inhibit the lipotoxicity of cerebral microvascular ECs (Nyunt et al., 2019). Another study also supports this view (Aung et al., 2016). Interestingly, however, another study reports opposite results, whereby after ATF3 gene knockout, the infarct size increased significantly in the mouse model of cerebral ischemia (Wang et al., 2012). ATF3 also inhibits the apoptosis of neurons and activation of microglia in the rat model of cerebral ischemia and alleviates ABI in rats (Ma et al., 2022). The study showed that compared to the SUMOylation mutant group, the SUMOylation degree and SUMO1-mediated expression of wild-type ATF3 increased significantly, thus accelerating the process of ECD induced by Ang II (Zhang et al., 2016). Simultaneously, ATF3 is a stress-induced adaptive response protein, which is associated with immune responses, tumors, inflammation, and other diseases (Zhou et al., 2011). ATF3 or SUMO1 gene knockout can inhibit the expression of inflammatory molecules induced by Ang II, while wild-type ATF3 can reduce the production of NO. Thus, SUMOylation of ATF3 can increase the stability of ATF3 and promote ECD mediated by angiotensin-converting enzyme II (Zhang et al., 2016).

The activation of focal adhesion kinase (FAK) can promote the progression of EC inflammation, and disturbed flow (D-flow) can induce EC senescence. D-flow can induce ROS production, further activating FAK through SUMOylation of FAK K152, forming a positive feedback loop, which induces EC activation and senescence. The site-directed mutation of FAK affecting SUMOylation reverses this phenomenon (Velatooru et al., 2021). Moreover, FAK mediates the expression of IL-6 in the brain and promotes inflammation (Keasey et al., 2018). Another study also showed that the mouse model of stroke had significant anti-inflammatory and neuroprotective effects after treatment with FAK inhibitors (Jia et al., 2022).

Extracellular signal-regulated kinase 5 (ERK5) is an anti-atherosclerotic factor (Heo et al., 2014), and its activation leads to the upregulation of PPAR, resulting in anti-inflammatory and anti-atherosclerotic effects (Woo et al., 2008). ERK5 can reduce its expression through the SUMOylation of PIAS1, thus accelerating the progression of inflammation (Paez-Mayorga et al., 2018). Moreover, in the case of hemodynamic abnormality, the expression of p53 and ERK5 is regulated by SENP2. In the mouse model carrying a SENP2 deletion, the degree of SUMOylation increased, and increased apoptosis and expression of ECs were observed, while in the SUMOylation mutation group, the apoptosis and expression of ECs were inhibited after overexpression of p53 and ERK5 (Heo et al., 2013). However, another study reports opposite conclusions. Ponatinib, a tyrosine kinase inhibitor (TKI), can upregulate SUMOylation of ERK5, promote the inflammatory progression of ECs, and disrupt the normal state of blood vessels (Paez-Mayorga et al., 2018). Protein kinase C ζ (PKC ζ) can induce SUMOylation of p53 and accelerate apoptosis of ECs (Heo et al., 2011; Dehnavi et al., 2019). A previous study suggests that increased expression of PKC ζ may play a role in preventing cognitive impairment in mice (Wu et al., 2012).

The regulatory effects of SUMOylation on endothelial cell inflammation show two completely opposite effects. Proinflammatory or anti-inflammatory effects are related to different molecules SUMOylation. However, it is uncertain whether such a role is beneficial or harmful. Considering the core role of ECs in NVU and BBB, the influence of SUMOylation on ECs may be an interesting research direction.

### 2.2 The regulatory effects of SUMOylation on neurons

The role of SUMOylation in neuroprotection has been studied for a long time now. After transient cerebral ischemia, the expression of SUMO2/3 in the hippocampus and cerebral cortex increases significantly, and the activation of the SUMOylation pathway can block the activation of oxidative stress responses and protect neurons (Yang et al., 2008). In the transgenic mouse model, increased expressions of SUMO1–3 were detected in the brain of mice after cerebral ischemia (Yang et al., 2014), however, SUMO1–3 gene knockouts showed limited neurological recovery after cerebral ischemia (Zhang et al., 2017). These studies show that overexpression of SUMO1/2 or Ubc9 can protect neurons while silencing SUMO2/3 is not conducive to neuronal survival (Datwyler et al., 2011; Lee et al., 2011; Zhang et al., 2019). The specific deletion of pericytes in SENP1 can enlarge the range of focal cerebral ischemia, aggravate motor dysfunction, and significantly increase neuronal damage after cerebral ischemia in mice. In the *in vitro* model, knockout of SENP1 from pericytes could activate the apoptosis process and destroy the integrity of the BBB (Sun et al., 2020). This is contrary to the conclusions of previous studies. A recent study has shown that the knockout of the SUMO2 gene in mouse neurons shows significant impairment in cognitive functions in mice. This process does not involve changes in gene structure and neuron morphology but is related to the impairment in synaptic SUMOylation (Yu et al., 2020). Another study using neural stem cells showed that mice with middle cerebral artery occlusion had strong hypoxia resistance after overexpression of SUMO E2 ligase (Ubc9), along with an enhanced differentiation ability of neurons *in vitro* (Bernstock et al., 2019). The expression of SUMOylation proteins increases significantly in the brain during coma. Subsequent *in vivo* and *in vitro* experiments have shown that brain SUMOylation can induce ischemic tolerance (Bernstock et al., 2018a), which can enhance the neuroprotective effect of SUMOylation by inhibiting the expression of SENP2 (Bernstock et al., 2018b).

Ion homeostasis involves almost all physiological and pathological processes, and sodium/calcium exchanger-3 (NCX3) plays a protective role after cerebral ischemia. A study on the model of cerebral infarction in rats shows that SUMO1 silencing aggravated brain injury after cerebral ischemia, and the combination of SUMO1 and NCX3 could enhance the neuroprotective effect after cerebral ischemia (Cuomo et al., 2016). In one study, the authors explored the therapeutic targets of neuronal apoptosis from the perspectives of biology and chemistry and found that SUMOylation played a neuroprotective role through drug activation (Krajnak and Dahl, 2018).

Hypothermic brain protection strategies have long been used clinically but their underlying molecular mechanism warrant investigation. After permanent occlusion of the middle cerebral artery in mice, hypothermic brain protection can greatly promote the level of SUMOylated proteins in the brain, thus protecting mice from ischemic injury (Lee et al., 2014). Hypoxia and hypothermia can promote SUMOylation of neural stem cells and resist hypoxia injury, while Ubc9 gene knockout can reverse this ability. SUMOylation is an important mechanism in neural stem cells for protection against hypoxia injury and can improve the survival rate and neural repair ability of neural stem cells after transplantation (Cai et al., 2022). Targeted temperature management (TTM) cools the whole body or target organs by lowering the temperature. TTM is of great significance in ischemia and reperfusion injury after ABI. TTM can induce SUMOylation, promote the regulation of unfolded protein responses, inhibit apoptosis and neuroinflammation, and plays a protective role in the brain (Talma et al., 2016).

With regard to the effects of SUMOylation on neurons, studies have shown that SUMOylation is more beneficial, but more work is needed to determine the exact effect of SUMOylation on neurons. In addition, the related mechanism is worthy of further study.

### 2.3 The regulatory effects of SUMOylation on microglia

Annexin-A1 SUMOylation regulates microglial polarization after cerebral ischemia by modulating the IκB kinase stability *via* selective autophagy and significantly improves the neurological functions in a mouse model of ischemic (Li et al., 2021). The expression of SENP6 in microglia increases significantly after cerebral ischemia, and after downregulation of SENP6 in microglia, positive results were obtained, whereby not only did the area of cerebral infarction reduce significantly but the motor and cognitive functions of mice with cerebral ischemia also improved substantially (Mao et al., 2022). However, opposite results have also been reported. Alcohol causes inflammation in the hippocampus of rats, which is caused by an increase in the number of microglia and is mediated by p-NF-κB-p65. The overexpression of SENP6 can inhibit this process, prevent the progression of neuroinflammation, and play a neuroprotective role (Li et al., 2019). Intermittent hypoxia can cause neuronal inflammation and neuronal apoptosis in mice, likely due to the downregulation of the expression of PPAR *γ* due to increased SUMOylation, and further downregulation of SENP1, together aggravating neuroinflammation and neuronal apoptosis (Wang et al., 2021b). Another study reported similar results. SENP1 overexpression inhibits inflammation caused by intermittent hypoxia, while SENP1 knockout leads to cognitive decline in mice (Wang et al., 2021c). Moreover, by regulating SUMOylation of NF- κB essential modulator (NEMO), overexpression of SENP1 can reduce the activation of NF- κB, thus inhibiting the inflammatory responses induced by microglia (Yang et al., 2020).

The effect of SUMOylation on microglia showed different results. SUMOylation may improve cognitive function through microglia, but it may also aggravate neuroinflammation. The difference in results may be due to different research methods, but it also suggests the diversity of SUMOylation in regulating the function of microglia. In view of the long-term pro-inflammatory effect of microglia on neuroinflammation, we should make more efforts in this research direction.

### 2.4 The regulatory effects of SUMOylation on astrocytes

Guanosine plays an active role in many diseases, especially neuroprotection (Lanznaster et al., 2016; Tasca et al., 2018). For example, guanosine has an anti-inflammatory effect during astrocyte senescence (Souza et al., 2016). Guanosine also has a neuroprotective effect on cerebral ischemia, which may be related to its ability to reduce the production of ROS (Dal-Cim et al., 2019). Guanosine can stimulate the proliferation of neural stem cells through the activation of CREB (Su et al., 2013). A recent study has shown that the neuroprotective effect of guanosine is related to the SUMOylation of neurons and astrocytes, and guanosine receptor antagonists can reverse this effect, suggesting that guanosine can play a role as a SUMOylation enhancer for neuroprotection (Zanella et al., 2020) (Table 1).

**TABLE 1:** The regulatory effect of SUMOylation on NVU composition.

Components	Genes/Pathways	Regulated pathways	Effects	References
Astrocytes	Guanosine	SUMOylation	Neuroprotection	Zanella et al. (2020)
Endothelial cells	Trx2	SUMOylation	Effectively inhibit the senescence of ECs induced by ROS	Chen et al. (2019)
	FAK K152	SUMOylation	Induces EC activation and senescence	Velatooru et al. (2021)
	GATA2	deSUMOylation	Aggravating endothelial inflammation	Qiu et al. (2017)
	NLRP3	SUMOylation	Inhibit the progression endothelial inflammation	Barry et al. (2018)
	ATF3	SUMOylation	Accelerating the process of ECD induced by Ang II	Zhang et al. (2016)
	ERK5	SUMOylation	Accelerating the progression of inflammation	Paez-Mayorga et al. (2018)
	p53 and ERK5	SUMOylation	Ancreased apoptosis and expression of ECs	Heo et al. (2013)
	PKC ζ	SUMOylation	Accelerate apoptosis of ECs	Heo et al. (2011); Dehnavi et al. (2019)
	PPAR *γ*	SUMOylation	Aggravated ECD	Kim et al. (2019); Yuan et al. (2019)
	MMP13	SUMOylation	Promotes angiogenesis	Yang et al. (2013)
	PAX6	deSUMOylation	Accelerate the repair of corneal ECs after injury	Yu et al. (2020)
Neurons	SUMO2/3	SUMOylation	Block the activation of oxidative stress responses and protect neurons	Yang et al. (2008)
	SUMO1/2	SUMOylation	Protect neurons	Datwyler et al. (2011); Lee et al. (2011)
	Ubc9	SUMOylation	Protect neurons	Zhang et al. (2019)
	SUMO2	deSUMOylation	Significant impairment in cognitive functions in mice	Yu et al. (2020)
	Ubc9	SUMOylation	Enhanced hypoxia resistance, enhanced differentiation ability of neurons *in vitro*	Bernstock et al. (2019)
	SENP2	SUMOylation	Enhanced the neuroprotective effect	Bernstock et al. (2018b)
Microglia	SENP6	SUMOylation	Not only did the area of cerebral infarction reduce significantly but the motor and cognitive functions of mice with cerebral ischemia also improved substantially	Mao et al. (2022)
	p-NF-κB-p65	deSUMOylation	Prevent the progression of neuroinflammation	Li et al. (2019)
	NEMO	deSUMOylation	Inhibiting the inflammatory responses induced by microglia	Yang et al. (2020)
Pericyte	SENP1	deSUMOylation	Enlarge the range of focal cerebral ischemia, aggravate motor dysfunction, and significantly increase neuronal damage after cerebral ischemia in mice	Sun et al. (2020)

## 3 Regulatory effects of SUMOylation on vascular remodeling

### 3.1 Effects of SUMOylation on vascular smooth muscle cells

VSMCs are the main components of the vascular wall, which play a key role in the stability of vascular structure and the maintenance of normal vascular pulsation (Lacolley et al., 2017). VSMCs are characterized by the expression of contractile proteins, including actin and myosin (Poittevin et al., 2014). VSMCs regulate CBF by contracting and relaxing blood vessels, changing the diameter of blood vessels, and regulating the supply of oxygen and nutrients (Hartmann et al., 2022). VSMCs are regulated by several molecular mechanisms, affecting the expression of related genes, thus changing vascular tension (Liu and Lin, 2022). The renin-angiotensin system (RAS) is the key medium for VSMCs to perform their physiological functions (Griendling et al., 1997), and the Notch signaling pathway regulates the migration and adhesion of VSMCs (Sorrentino et al., 2022). VSMCs mainly include two phenotypes. Contractile VSMCs function mostly when blood vessels are healthy, and switch to synthetic VSMC phenotype after vascular damage (Rensen et al., 2007; Touyz et al., 2018). The changes in VSMCs are related to cell signaling proteins, injury stimulation, and cell-to-cell contact. After a neurovascular injury, VSMCs dedifferentiate and promote vascular repair (Davis-Dusenbery et al., 2011). Generally, normal VSMC function is very important for the maintenance and repair of neurovascular function, and VSMC dysfunction leads to the progression of vascular disease.

In the biological process underlying VSMC proliferation, Kruppel-like factor 4 (KLF4) acts as a switch (Nie et al., 2016). When the reaction between KLF4 and the SUMO-binding enzyme, Ubc9 is inhibited, the proliferation of VSMCs decreases. Another study reports a similar conclusion (Li et al., 2018). In addition, peroxisome proliferator-activated receptors (PPARs), an important regulator of lipid metabolism, also play an important role in the proliferation of VSMCs. SUMOylation of PPAR *γ* one promotes the proliferation and migration of VSMCs (Lim et al., 2009; Wadosky and Willis, 2012). The Rho-specific guanine nucleotide dissociation inhibitor (RhoGDI) can regulate the proliferation of VSMCs and the stability of the thrombus. When Ang II receptor one is activated, Ang II can increase the stability of RhoGDI and promote the proliferation of VSMCs through SUMOylation (Dai et al., 2019). Myocardin is a factor regulating the differentiation of smooth muscle cells (SMCs), which can be modified by SUMO1 to increase protein stability, and this process is reversibly regulated by SENP2, which is known to promote the phenotypic switching of VSMCs (Liang et al., 2022).

Autophagy is a research hotspot, and its activation is related to the proliferation of VSMCs under hypoxic stress (Wen et al., 2019). In the mouse model of hypoxic pulmonary hypertension, the overexpression of SUMO1 activates the autophagy pathway, resulting in the dedifferentiation of VSMCs from the pulmonary artery and enhanced VSMC proliferation (Yao et al., 2019). In hypoxic mice, the expression of SUMO1 is upregulated, and a significant increase in VSMC proliferation, migration, dedifferentiation, and autophagy is observed. Downregulation of SUMO1 expression can reverse these phenotypes (Yao et al., 2019). On the contrary, another study concluded that HIF-1 α could increase the expression of the downstream gene, VEGF, and enhance the proliferation of pulmonary artery smooth muscles in rats through SENP1-mediated deSUMOylation (Zhou et al., 2016). These two studies on pulmonary artery smooth muscles have shown opposite results. Thus, evaluating the role of SUMOylation in cerebral VSMCs is a potential research direction.

As a SUMOylation proteolytic enzyme, SENP3 plays an important role in the occurrence and development of many tumors (Han et al., 2010). Studies have shown that a high expression of SENP3 is closely related to arterial remodeling. It can significantly promote the proliferation and migration of VSMCs while silencing SENP3 reverses this effect and slows down vascular remodeling in mice. In addition, increased SENP3 expression can also enlarge the area of the remodeled arterial intima (Cai et al., 2021). Another study showed that SUMOylation had a positive effect on VSMCs, increasing vascular intimal thickness and muscle fiber production in mice, all promoting vascular remodeling (Dai et al., 2019).

The proliferation of VSMCs is very important for the stability of normal vascular structure. Related studies have shown that SUMOylation can promote the proliferation and migration of VSMCs, but there are different research conclusions. Generally speaking, the role of SUMOylation on VSMCs can not be ignored and needs to be further explored.

### 3.2 Effects of SUMOylation on angiogenesis

Angiogenesis plays a key role in various physiological and pathological processes, including inflammation, diabetes, infection, and cancer. Angiogenesis means to regenerate and maintain the structure of blood vessels based on the original blood vessels, which is a dynamic and complex process (Carmeliet and Jain, 2011). Blood vessels originate from endothelial progenitor cells through a process called vasculogenesis, and further collateral circulation formation occurs, referred to as angiogenesis (Carmeliet, 2003). After ABI, angiogenesis helps restore the supply of oxygen and nutrients to the damaged brain, stabilize blood perfusion in the brain, maintain the survival of neurons, and promote the recovery of the nervous system. Research on VEGF1–5 and their receptors, placental growth factors (PLGFs), fibroblast growth factors (FGFF1, FGFF2) and their receptors, transforming growth factors, and tumor necrosis factor has been conducted. They are angiogenic factors and anti-angiogenic factors (Huang and Bao, 2004; Ucuzian et al., 2010). There are many sources of angiogenic factors, including ECs, fibroblasts, platelets, and cancer cells (Ucuzian et al., 2010).

VEGFs mediate physiological processes mainly by activating VEGFR2, which participates in cell migration, regulation of endothelial connections, and angiogenesis (Simons et al., 2016). During the process of pathological angiogenesis in VEGFR2, the loss of SENP1 hinders pathological angiogenesis and tissue repair and reduces VEGF-induced angiogenesis (Zhou et al., 2018). SUMOylation not only regulates PTMs of proteins but also regulates VEGFR at the gene level. Related studies have shown that PROX1, which regulates lymphangiogenesis, is also regulated by SUMOylation when it induces VEGFR expression (Pan et al., 2009). Basic FGF (FGF2) is a pro-angiogenic factor, which can activate the FGF receptor-1 (FGFR1) of ECs to promote angiogenesis. Under hypoxia, FGFR is SUMOylated, which promotes VEGF2 aggregation but limits VEGF1 aggregation, thus activating the VEGFR2 signal and enhancing angiogenesis (Zhu et al., 2022). However, some studies have shown that VEGFR-2 can specifically bind to SUMO1, inhibit the angiogenic signal pathway in non-small cell lung cancer cells, and prevent the malignant progression of tumor cells (Wang and Jiang, 2020).

The notch signaling pathway plays an important role in many physiological and pathological processes, as well as vascular diseases. The activation of the Notch1 pathway in ECs can inhibit the expression of VEGFR, restrict the transmission of the VEGF signaling axis, and reduce angiogenesis (Benedito et al., 2009). The absence of SENP1 in ECs leads to long-term SUMOylation of the Notch1 signaling pathway and slows down the speed of retinal vascularization. Notch1 can reversibly regulate signal transduction for endothelial angiogenesis through SUMOylation (Zhu et al., 2017). In neurovascular diseases, the regulatory role of SUMOylation on Notch pathway deserves further exploration.

In a study on cerebral ischemic stroke, inhibition of the AKT/mTOR pathway was found to promote angiogenesis and neurogenesis and improve CBF and glucose metabolism (Zhao et al., 2019). However, the opposite results have been obtained in a study on cardiovascular disease. *In vitro* and *in vivo* experimental studies have shown that SENP2 gene deficiency can improve cardiac function after myocardial infarction in mice owing to the increase in the SUMOylation activity of targeting protein kinase B (AKT), which promotes cardiomyocyte proliferation and angiogenesis (Chen et al., 2021). In another study, similar results were obtained. A decrease in the level of SUMOylation reduced AKT activation and cell proliferation. Interestingly, the activation of AKT is not regulated by the classical PI3K pathway (de la Cruz-Herrera et al., 2015). A study on cardiac ischemia-reperfusion injury showed that after the injury, the activity of the deSUMOylation enzyme decreased, while some genes related to angiogenesis showed SUMOylation changes, indicating its important role in the process of cardiac angiogenesis (Hotz et al., 2020). The difference in the regulation of AKT in cardio-cerebral angiogenesis may be the potential research direction of assessing the role of SUMOylation on the regulation of neuroangiogenesis.

A recent study showed that Astragaloside IV (AS-IV) could stabilize HIF-1 α protein by activating the SUMOylation pathway, and could promote the proliferation and migration of VECs, thus improving angiogenesis under hypoxic conditions and accelerating wound healing (Wang B. et al., 2021). SUMOylation is also closely related to angiogenesis in tumor-related research. For example, angiogenesis under hypoxia is promoted in prostate cancer cells, which is regulated by androgen receptor-dependent SUMOylation (Vlachostergios and Papandreou, 2012).

## 4 Conclusion and perspective

ABI remains a major health problem, posing a heavy burden on these patients and their families. Many studies have focused on the treatment of ABI, including targets for molecular therapy and drug development, and some promising research directions have been applied in clinical settings. However, considering the complexity of ABI and individual differences among patients, the effectiveness of monotherapy cannot be exaggerated, because a single treatment cannot solve pathological progression.

In this review, we briefly report the composition and normal physiological functioning of the neurovasculature. This paper focuses on the role of SUMOylation in phenotypes related to neurovascular pathological progress, including EC disturbance, VSMC proliferation, angiogenesis, *etc.* We also discussed the neuroprotective effects of SUMOylation. For easier elaboration, we wrote the above sections separately but in reality, physiological and pathological processes are not strictly independent but occur almost simultaneously and involve mutual integration. For example, the improvement in EC function also has a positive effect on angiogenesis; SMC proliferation is also a part of vascular remodeling. ECs are not only the basis of VSMC proliferation, angiogenesis, and vascular remodeling but also many scholars of several fields, resulting in the accumulation of research results, so we focused on this aspect in the review. However, its limitation is that we only reviewed the correlation between SUMOylation and ABI, and did not detail its internal relationship and causal relationship.

Remarkable progress has been made in the study of SUMOylation and ABI worldwide. However, given the complexity of the pathological mechanism of ABI, the development of treatment is still challenging. The purpose of this review is to understand how neurovascular disorders associated with ABI occur; how SUMOylation is involved in these injuries, and how SUMOylation improves ABI. It is important to understand the role of SUMOylation in ABI as it may be the potential molecular mechanism underlying ABI or brain protection. We hope that this review will provide theoretical references for future research on SUMOylation and ABI.
